# A Complete Encyclopædia of Medical Jurisprudence

**Published:** 1839-04

**Authors:** 


Art. VI.—*Ausfürliche Encyklopädie der gesammten Staatsarzneikunde.* Von Georg Friederich Most, Doctor der Philosophie, Medicin, und Chirurgie, &c. 1stes Heft.—*Leipzig*, 1838. pp. 192.

*A Complete Encyclopædia of Medical Jurisprudence.* By Dr. George F. Most. First Part.—*Leipzig*, 1838. pp. 192.

Dr. Most has already acquired some reputation as editor of an Encyclopædia of Medicine and Surgery. We hardly expected that medical jurisprudence would have so soon found itself in an encyclopedic form; nor are we satisfied that the plan thus adopted by Dr. Most will render this work so practically useful as he evidently intends it to be. Our opinion is founded on the fact that in legal medicine, more perhaps than in any other branch of science, it is difficult to determine under what head to arrange the numerous and complicated questions to which the different subjects give rise. Unless, then, the whole of the work be read through and studied, it is not likely to become one of easy reference for the solution of numerous important questions. Nevertheless, we are extremely well pleased with the contents of this first part. Although extending over only 192 pages, it contains, owing to the smallness of type, as much or even more matter than numerous large octavos. A great number of interesting subjects are here discussed. Some of the larger articles have the names of the authors attached to them. The subjects of Abortion, Contagion, Arsenic, and Medical Jurisprudence, including a history of the science and its literature, are ably handled. There is also a great deal of practical toxicology, chiefly extracted from Gusserow's treatise, which we noticed in one of our late Numbers. The processes for detecting the oxalic and hydrocyanic acids are well detailed; but it is surely an error to assert that the latter does not redden litmus paper, unless it contain sulphuric acid. Each article is accompanied by numerous references; and, in general, the synonyms of foreign languages are placed at the head of it. We are at a loss to conceive where the editor could have learnt that the science was called "Juridical Physick" in this country, or that the practitioner of it was known as a "juridical physician." This Encyclopædia embraces all the subjects of medical police, and numerous others in the circle of the medical sciences, in so far as they have the least reference to the object of this work. We trust that the future Numbers will be as rich in information as the first.

